# Radiomics assessment of carotid intraplaque hemorrhage: detecting the vulnerable patients

**DOI:** 10.1186/s13244-022-01324-2

**Published:** 2022-12-20

**Authors:** Shuai Zhang, Lin Gao, Bing Kang, Xinxin Yu, Ran Zhang, Ximing Wang

**Affiliations:** 1 The School of Medicine, Shandong First Medical University, No. 6699, Qingdao Road, Huaiyin District, Jinan, China; 2 Department of Radiology, Shandong Provincial Hospital Affliated to Shandong First Medical University, No. 324 Jingwu Road, Jinan, 250021 China; 3 Huiying Medical Technology Co. Ltd., 66 Xixiaokou Road, Haidian District, Beijing, China

**Keywords:** Radiomics, Atherosclerosis, Carotid artery, Intraplaque hemorrhage, Computed tomography

## Abstract

**Background:**

Intraplaque hemorrhage (IPH), one of the key features of vulnerable plaques, has been shown to be associated with increased risk of stroke. The aim is to develop and validate a CT-based radiomics nomogram incorporating clinical factors and radiomics signature for the detection of IPH in carotid arteries.

**Methods:**

This retrospective study analyzed the patients with carotid plaques on CTA from January 2013 to January 2021 at two different institutions. Radiomics features were extracted from CTA images. Demographics and CT characteristics were evaluated to build a clinical factor model. A radiomics signature was constructed by the least absolute shrinkage and selection operator method. A radiomics nomogram combining the radiomics signature and independent clinical factors was constructed. The area under curves of three models were calculated by receiver operating characteristic analysis.

**Results:**

A total of 46 patients (mean age, 60.7 years ± 10.4 [standard deviation]; 36 men) with 106 carotid plaques were in the training set, and 18 patients (mean age, 61.4 years ± 10.1; 13 men) with 38 carotid plaques were in the external test sets. Stenosis was the independent clinical factor. Eight features were used to build the radiomics signature. The area under the curve (AUC) of the radiomics nomogram was significantly higher than that of the clinical factor model in both the training (*p* = 0.032) and external test (*p* = 0.039) sets.

**Conclusions:**

A CT-based radiomics nomogram showed satisfactory performance in distinguishing carotid plaques with and without intraplaque hemorrhage.

**Supplementary Information:**

The online version contains supplementary material available at 10.1186/s13244-022-01324-2.

## Key points

- Distinguishing carotid plaques with intraplaque hemorrhage is difficult by conventional imaging.
- A radiomics nomogram facilitates differentiation of carotid plaques with intraplaque hemorrhage.
- CT-based nomogram may be a non-invasive tool to identify carotid plaque vulnerability.

**Supplementary Information:**

The online version contains supplementary material available at 10.1186/s13244-022-01324-2.

## Background

In several guidelines and clinical practice, the prevention of stroke in patients with carotid plaques is based on severity of luminal narrowing [1, 2]. However, with the advances in imaging techniques, there has been renewed interest in characteristic and detection of the features of carotid plaque vulnerability beyond luminal narrowing [3]. Intraplaque hemorrhage (IPH), one of the key characteristics of vulnerable plaques, has been demonstrated to be closely related to incidence of stroke independence of the degree of stenosis [4, 5]. Most studies showed that several imaging methods can be used as a non-invasive way to evaluate plaque vulnerability and detect the IPH. MRI is considered the best imaging technique for identification of IPH with high specificity and sensitivity with histology as the gold standard [6]. However, patients with claustrophobia and those who have been placed with metal stent cannot undertake MRI. CTA is a valuable imaging modality for pretreatment evaluation of atherosclerotic patients due to its speed, high spatial resolution, and accessibility. But the detection of IPH is difficult by conventional CT imaging and more dependent on the experience of radiologists.

Radiomics can use computer data mining techniques to obtain quantitative, valuable information that cannot be obtained from assessing routine imaging by physician. [7]. Radiomics analysis provides us with a quantitative method to reflect tissue heterogeneity and is more reliable compared with subjective evaluation [8]. The studies of radiomics analysis for plaque evaluation have emerged recently but until now are relatively rare compared to oncology [9–11]. Recently, CT- and MRI-based radiomics analysis has been used to identify vulnerable carotid plaques [9, 10]. And previous studies have also shown that radiomics analysis of plaque texture on MRI can distinguish between symptomatic and asymptomatic basilar plaques [11]. To our knowledge, no relevant studies have been performed to detect the IPH in carotid arteries by CT-based radiomics approach.

Therefore, in our study, our purpose was to develop and validate a CT-based radiomics nomogram incorporating clinical factors and a radiomics signature for the detection of IPH in carotid arteries.

## Methods

### Study population

**Fig. 1:**
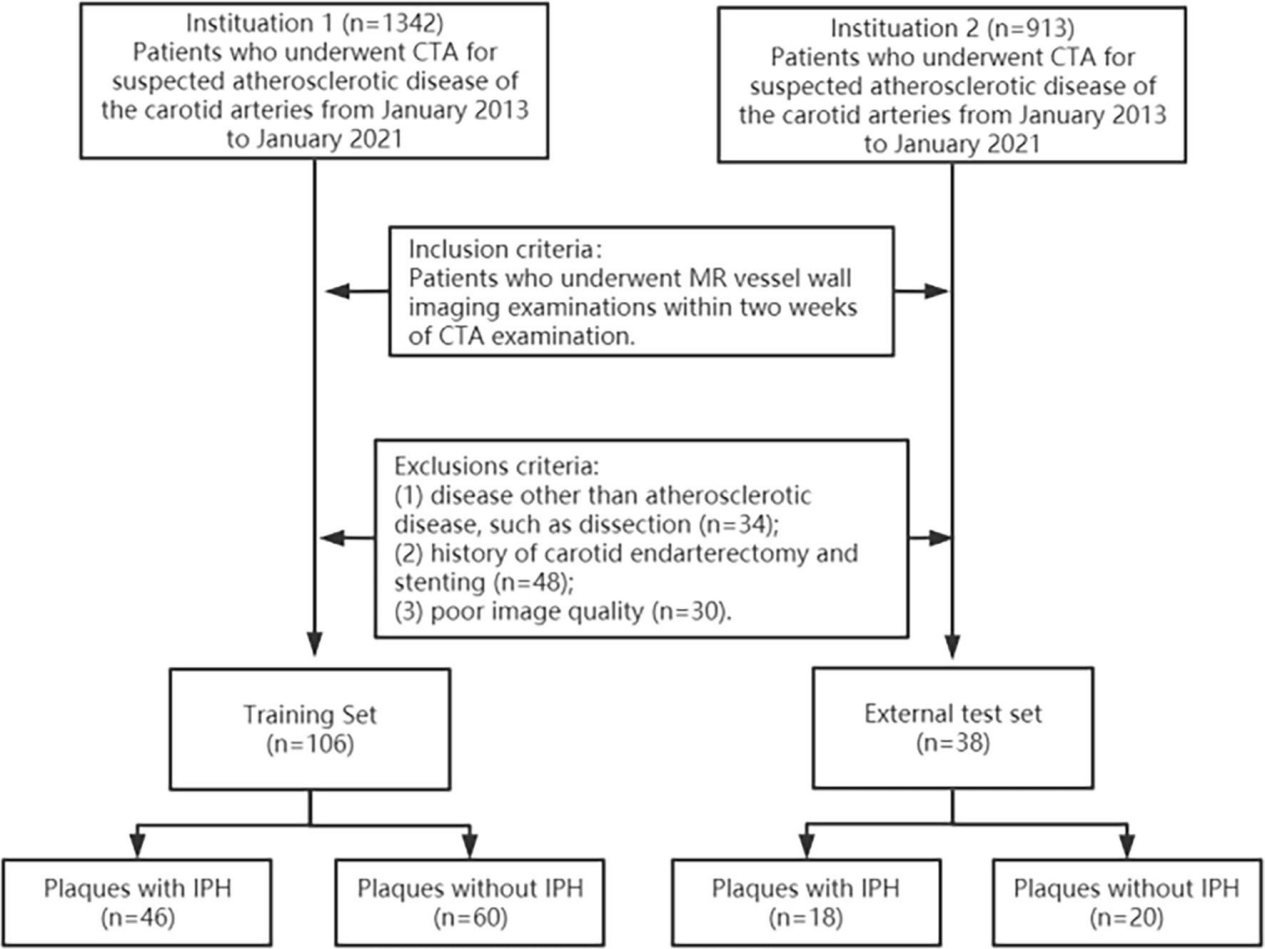
Flow diagram of the study. IPH = intraplaque hemorrhage

Institutional review board approval was obtained for all study procedures, and informed consent was waived because of the retrospective nature of the study. We screened consecutive patients who underwent CTA for suspected atherosclerotic disease of the carotid arteries from January 2013 to January 2021 in Shandong Provincial Hospital Affiliated to Shandong First Medical University and Shandong Medical Imaging Research Institute. The inclusion criterion was patients who underwent MR vessel wall imaging examinations within two weeks of CTA examination. Exclusions criteria were as follows: (1) disease other than atherosclerotic disease, such as aneurysm; (2) poor image quality; and (3) history of carotid stenting and endarterectomy. Flowchart for selecting the study population is shown in Fig. 1.

### MRI image acquisition

The high-resolution MRI was performed on a 3.0 Tesla MRI scanner (Prisma, Siemens Healthineerse; Ingenia, Philips Healthcar; Verio, Siemens Healthineers) with a standard 64-channel head-neck coil. The MRI protocols are described in Additional file 1: Table S1.

### CTA protocol

CTA examination was performed on multi-slice CT scanners (SOMATOM Force, Siemens Healthineers; SOMATOM Definition Flash, Siemens Healthineers). A 60–70 mL volume of contrast agent (Omnipaque-350; GE Healthcare) was injected at the speed of 5 mL/s, and then followed by 50 mL of saline flush, with a electric injector. After the aortic arch reached the attenuation threshold of 100 Hounsfield units (HU) for 5 s, bolus tracking was used to trigger the acquisition. The carotid CTA scanning parameters of all scanners were as follows: tube voltage of 100 kVp, reconstructed slice thickness of 0.5 mm, reconstructed slice interval of 0.5 mm, pitch of 1.0 and rotation time of 350 ms. Scanning range was from the aortic arch to skull vertex.

### Image analysis

Carotid IPH was defined that the presence of higher signal intensity (at least 1 voxel showing 1.5 times higher signal intensity compared to adjacent sternocleidomastoid muscle) was seen on the T1-weighted fat-saturated turbo spin echo [6]. The measurements of CTA markers were obtained by using post-processing workstation (Syngo.via, Siemens Force, Germany). The degree of stenosis of carotid arteries was defined on CTA according to the North American Symptomatic Carotid Endarterectomy Trial criteria [12]. Plaque ulceration was determined as the existence of at least 2 mm of contrast agent protruding into the plaque on any single plane [13].

### Development of clinical factor model

Univariable analysis was applied to compare the differences in clinical factors between the two groups. Then, a multiple logistic regression analysis was applied to construct the clinical factor model by using the significant variables from the univariable analysis as inputs. Odds ratios (OR) as estimates of relative risk with 95% confidence intervals (CI) were calculated for each independent factor.

### Segmentation of plaque images and radiomics feature extraction

Regions of interests (ROIs) were manually segmented in the segmentation and feature extraction were performed with a postprocessing platform (Huiying Medical Technology Co., Ltd) cross-sectional area of the plaque. Contouring was drawn within the border of the plaque, and the adjacent normal tissues were not covered. Two radiologists (B.K. and G.H., with 7 and 8 years of experience in vascular radiology, respectively) independently performed the ROI segmentation. In order to eliminate the influence of dimension between features and make the intensity information consistent, the image was normalized before analysis, which eliminated the interference caused by different CT equipment manufactures.

### Development of radiomics signature and radiomics nomogram

**Fig. 2:**
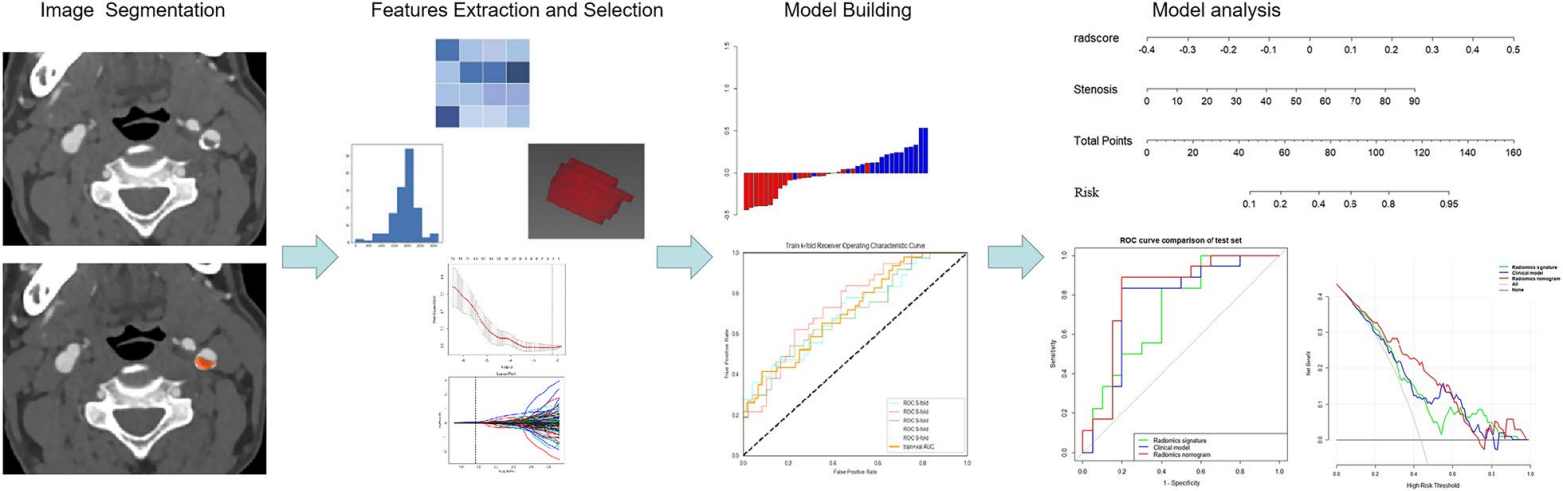
Overall workflow of the radiomics model development

The radiomics features that met the criteria of having intraclass correlation coefficients (ICCs) greater than 0.75 were tested by one-way analysis of variance (ANOVA) to select important features. The remaining features were then included in select_k_best method and least absolute shrinkage and selection operator (LASSO) regression model to choose the most valuable features in the training cohort. Then, the selected features were applied to compose a radiomics signature. Fivefold cross-validation was performed by iterating over feature selection and model development for each subset. A radiomics score (Rad-score) was calculated. A radiomics nomogram was constructed by combining the radiomics signature and the significant variables of the clinical features. The overall workflow of the radiomics model development is displayed in Fig. 2.

### Assessment of the performance of three models

The diagnostic performance of the clinical factor model, the radiomics signature, and the radiomics nomogram for identification of IPH in carotid arteries was assessed from the area under curve (AUC) of the receiver operator characteristic (ROC) curve in both the training and validation sets. To evaluate the clinical practicability of nomogram, a decision curve analysis (DCA) was carried out by calculating the net benefits.

### Statistical analysis

Univariable analysis was used to compare differences in the clinical factors between the two patient groups, with independent samples t tests for quantitative data, and chi-square or Fisher’s exact tests for qualitative data, as appropriate. One-way ANOVA was performed to compare the values of each radiomics feature for the differentiation of carotid plaques with and without IPH. Differences in the AUC values among three models were evaluated by Delong test. Statistical significance was considered at *p* < 0.05. Statistical analysis was performed with SPSS (version 22.0, IBM) and R statistical software (version 3.3.3, https://www.r-project.org).

## Results

### Clinical factors of the patients

Thirty-four patients who had a carotid dissection, forty-eight patients who had previously undergone carotid endarterectomy and stenting, and thirty patients who had poor image quality were excluded. A total of 46 patients (mean age ± standard deviation, 60.7 ± 10.4 years; 36 men) with 106 carotid plaques from Shandong Provincial Hospital Affiliated to Shandong First Medical University comprised the training set. An external validation set contained 18 patients (mean age, 61.4 ± 10.1 years; 13 men) with 38 carotid plaques from Shandong Medical Imaging Research Institute.

The clinical characteristics of the patients in the training and external test sets are summarized in Table 1. Degree of luminal stenosis, maximum plaque thickness, and ulceration showed significant differences between the plaques with IPH and plaques without IPH (*p* < 0.05) in training set. The multiple logistic regression analysis showed that stenosis (OR 10.4; 95% CI 1.01–1.07; *p* = 0.008) remained as independent predictors in the clinical factor model.

**Table 1:** Clinical factors of the training and validation sets

Clinical factors	Training set (*n* = 106)			External test set (*n* = 38)		
	Plaques with IPH (*n* = 46)	Plaques without IPH (*n* = 60)	*p*	Plaques with IPH (*n* = 18)	Plaques without IPH (*n* = 20)	*p*
Age, y	65.4 ± 9.4	62.0 ± 8.1	0.55	63.7 ± 9.4	65.0 ± 8.0	0.653
Sex, male	39 (84.8%)	47 (78.3%)	0.460	12 (66.7%)	18 (90.0%)	0.117
BMI, kg/m^2^	25.9 ± 1.4	26.5 ± 1.8	0.89	26.6 ± 1.4	26.4 ± 1.3	0.747
Hypertension	34 (73.9%)	50 (83.3%)	0.334	16 (88.9%)	7 (35.0%)	0.001
Hyperlipidemia	23 (50.0%)	27 (45.0%)	0.696	13 (72.2%)	8 (40.0%)	0.058
Diabetes	17 (37.0%)	17 (28.3%)	0.404	3 (16.7%)	0 (0%)	0.097
Smoking	25 (54.3%)	31 (51.7%)	0.846	11 (61.1%)	17 (85.0%)	0.144
CAD	24 (52.2%)	21 (35.0%)	0.112	11 (61.1%)	7 (35.0%)	0.193
Antihypertension use	29 (63.0%)	33 (55.0%)	0.433	15 (83.3%)	4 (20.0%)	< 0.001
Statin use	25 (54.3%)	31 (51.7%)	0.846	8 (44.4%)	7 (35.0%)	0.741
Antiplatelet use	21 (45.7%)	31 (51.7%)	0.562	9 (50.0%)	11 (55.0%)	0.758
Calcification	36 (78.3%)	51 (85.0%)	0.447	12 (66.7%)	15 (75.0%)	0.724
Degree of stenosis, %	48.5 ± 19.5	32.6 ± 15.8	< 0.001	40.3 ± 21.8	36.4 ± 16.5	0.540
Maximum thickness, mm	4.0 ± 1.6	2.9 ± 1.0	< 0.001	4.2 ± 1.3	2.7 ± 0.9	< 0.001
Ulceration	6 (13.0%)	0 (0%)	0.005	3 (16.7%)	0 (0%)	0.097

Continuous variables are described as mean ± standard deviation, and categorical variables are presented as numbers (%)

*BMI* Body mass index, *CAD* Coronary artery disease, *IPH* Intraplaque hemorrhage

### Feature extraction, selection, and radiomics signature establishment

Among 1409 radiomics features extracted from CTA images, 946 features met the standard of an inter-observer and intra-observer ICCs greater than 0.75. A total of 602 radiomics features showing significant differences between plaques with and without IPH on one-way ANOVA were performed the select_k_best method to eliminate the redundant and irrelated features. These features were then passed through the LASSO to select the most valuable ones; 8 features for constructing the radiomics signature were finally selected by LASSO. These features were included in the Rad-score calculated as follows: Radscore = wavelet-HLL_gldm_DependenceVariance × 0.068140989 + wavelet-HLH_glcm_Imc1 × 0.022492042 - wavelet-LHH_ngtdm_Strength × 0.008320669 - wavelet-HLL_glcm_Imc2 × 0.023842802 + wavelet-HHL_firstorder_Variance × 0.035072963 + wavelet-HLH_firstorder_Kurtosis × 0.030005675 + exponential_firstorder_Variance × 0.008876562 + wavelet-LHL_firstorder_Variance × 0.014908661. The more details of the radiomics features can be found in Additional file 1: Table S2. A significant difference was found in the Rad-score between plaques with and without IPH in the training set (0.67 ± 0.13 vs. − 0.05 ± 0.12; *p* < 0.001), which was then confirmed in the external test set (0.19 ± 0.17 vs. − 0.16 ± 0.18; *p* < 0.001).

### The radiomics nomogram establishment and evaluation of the performance of three models

**Fig. 3:**
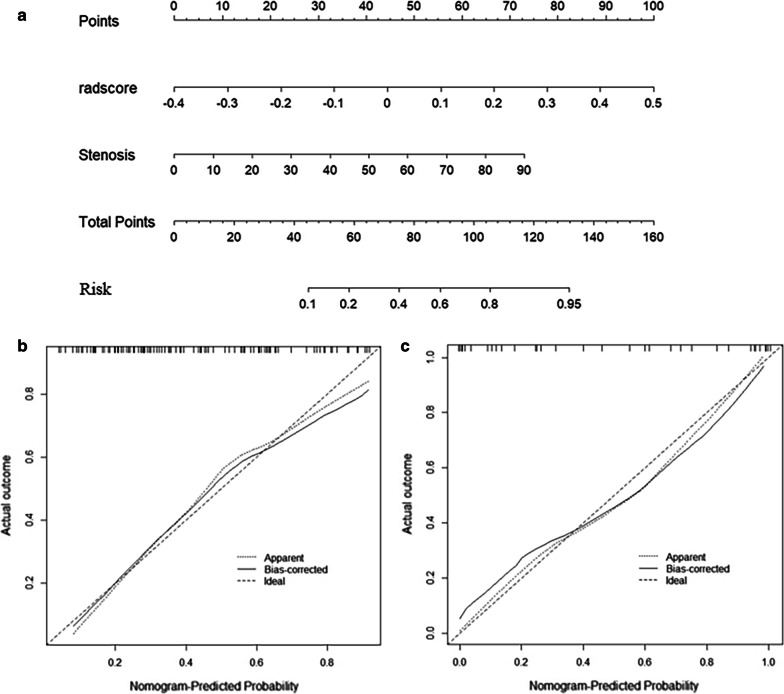
The radiomics nomogram and calibration curves for the radiomics nomogram. **a** The radiomics nomogram, combining stenosis and Rad-score, developed in the training set. Calibration curves for the radiomics nomogram in the training (**b**) and external test sets (**c**). Calibration curve indicates the goodness-fit for the nomogram. The 45° dotted line represents an ideal prediction, and the other dotted line represents the predictive performance of the nomogram. A closer distance between two lines indicates better prediction. The solid line represents the bias corrected

The Rad-score and stenosis were incorporated into a radiomics nomogram (Fig. 3a). Figure 3b, c shows the calibration curve of the nomogram. The calibration curve and the Hosmer–Lemeshow test showed good calibration in the training set (*p* > 0.05) and external test set (*p* > 0.05).

**Fig. 4:**
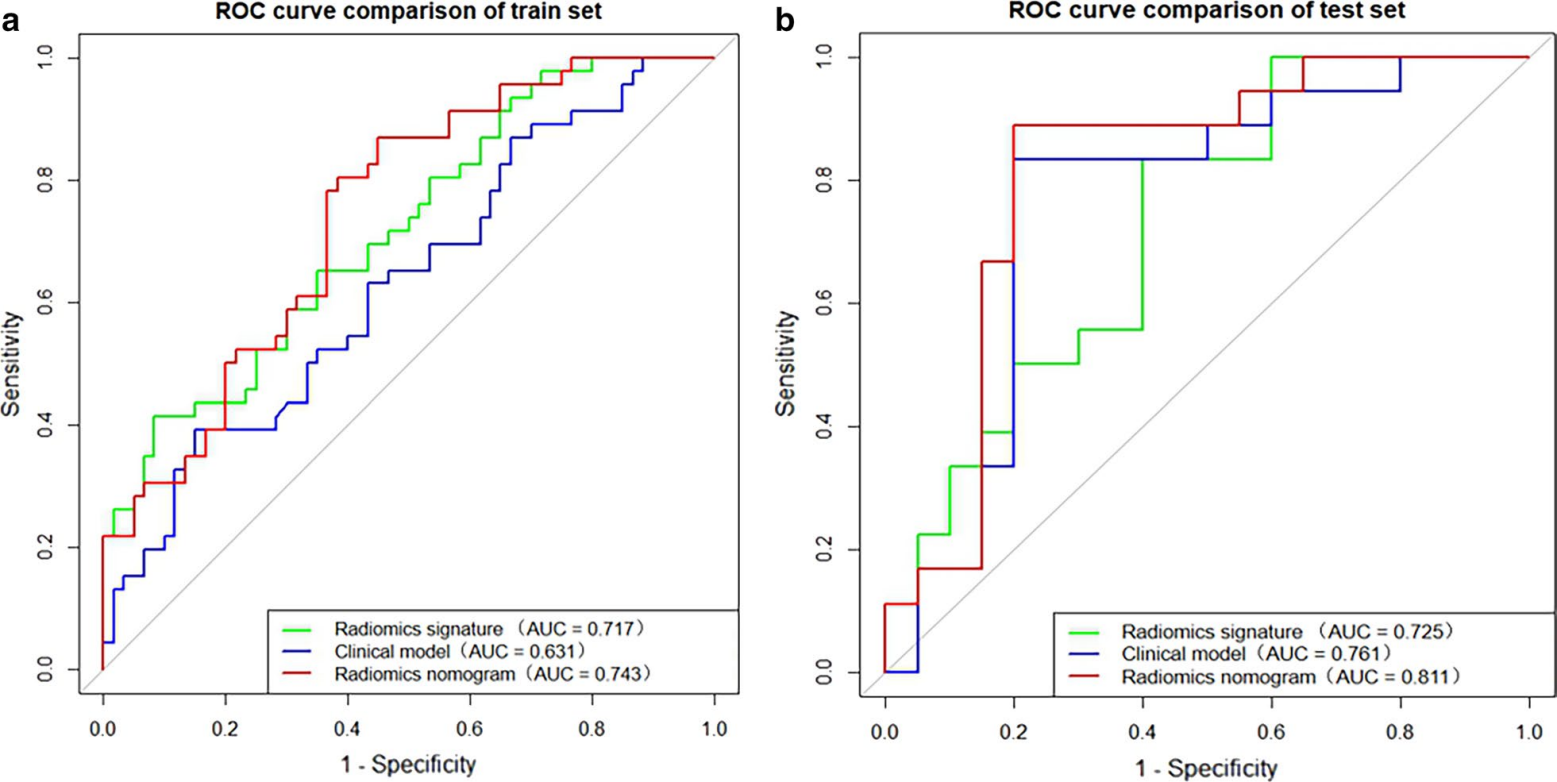
Receiver operating characteristic curves of the clinical factor model, the radiomics signature, and radiomics nomogram in the training (**a**) and external test (**b**) sets, respectively

The diagnostic performances of the clinical factor model, radiomics signature, and radiomics nomogram are summarized in Table 2. The ROC curves of the three models are shown in Fig. 4 for both the training and external test sets. The area under the curve (AUC) of the radiomics nomogram (AUC, 0.743; 95% confidence interval [CI], 0.650–0.835) was higher than that of the clinical factor model (AUC, 0.631; 95%CI, 0.524–0.738) in the training (*p* = 0.032). In the validation set, the radiomics nomogram (AUC, 0.811; 95%CI, 0.661–0.961) performed better (*p* = 0.039) than the clinical factor model (AUC, 0.761; 95%CI, 0.596–0.927). In the validation set, the sensitivity, specificity, and accuracy, respectively, were 62.5% (11 of 18 patients), 69.2% (14 of 20 patients) and 63.2% (24 of 38 patients) for radiomics signature model; 80.0% (14 of 18 patients), 77.8% (16 of 20 patients) and 78.9% (30 of 38 patients) for clinical model; and 88.9% (16 of 18 patients), 80.0% (16 of 20 patients) and 84.2% (32 of 38 patients) for radiomics nomogram.

**Table 2:** Diagnostic performance of the clinical factor model, radiomics signature, and radiomics nomogram for detection of intraplaque hemorrhage

Set	Model	AUC (95%CI)	Sensitivity (%)	Specificity (%)	Accuracy (%)
Training set	Clinical model	0.631 (0.524–0.738)	61.8 (28/46)	49.0 (29/60)	56.7 (60/106)
	Radiomics signature	0.717 (0.620–0.814)	68.9 (32/46)	60.0 (36/60)	65.1 (69/106)
	Radiomics nomogram	0.743 (0.650–0.835)	69.1 (32/46)	56.9 (34/60)	63.2 (67/106)
External test set	Clinical model	0.761 (0.596–0.927)	80.0 (14/18)	77.8 (16/20)	78.9 (30/38)
	Radiomics signature	0.725 (0.562–0.888)	62.5 (11/18)	69.2 (14/20)	63.2 (24/38)
	Radiomics nomogram	0.811 (0.661–0.961)	88.9 (16/18)	80.0 (16/20)	84.2 (32/38)

*AUC* Area under the curve, *CI* Confidence interval

**Fig. 5:**
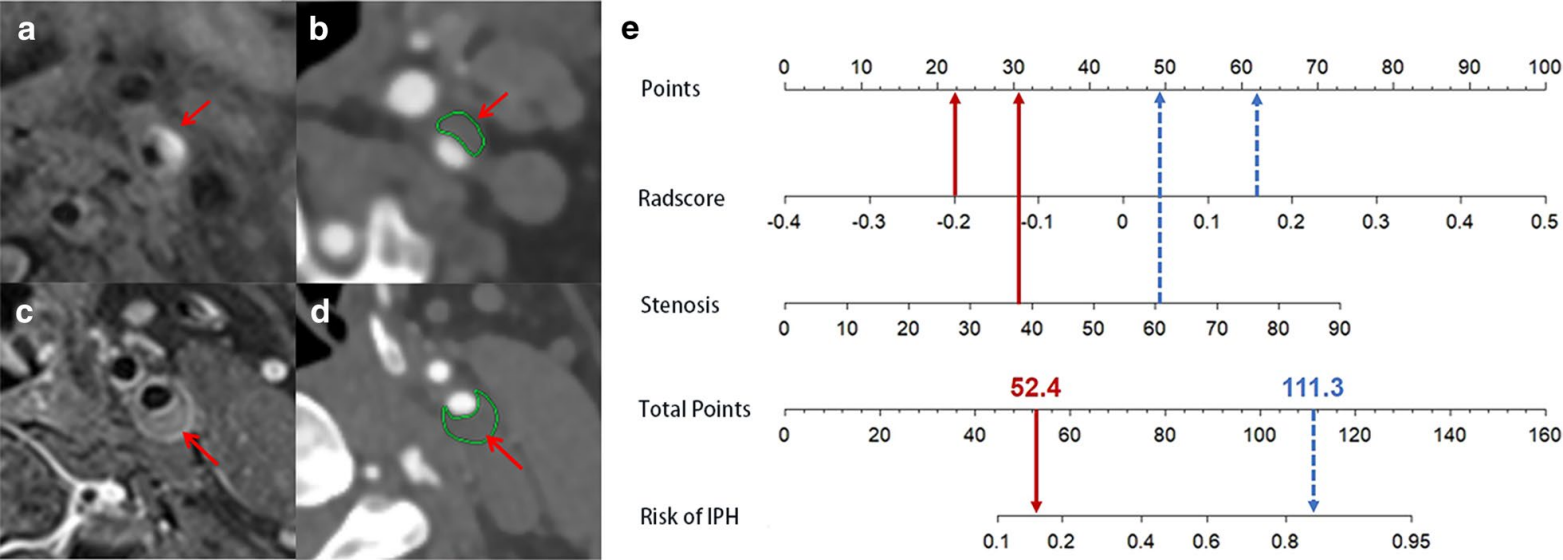
Application of nomogram to predict probability of IPH. **a**, **b** The carotid plaque (arrow) is shown on MRI and CTA. And MRI shows hyper-intensity on T1-weighted imaging suggestive of IPH. **c**, **d** The carotid plaque (arrow) is shown on MRI and CTA. And MRI shows no hyper-intensity on T1-weighted imaging suggestive of no IPH. **e** For patient in **a** and **b** (blue dashed arrows), nomogram yields total of 111.3 points and corresponding risk of IPH greater than 0.8. For patient in **c** and **d** (red solid arrows), nomogram yields total of 52.4 points and corresponding risk of IPH less than 0.2. IPH = Intraplaque hemorrhage

The DCA of three models is shown in Additional file 1: Fig. S1. The DCA showed that the overall net benefit of the radiomics nomogram in differentiating carotid plaques with IPH and without IPH plaques was higher than that of the clinical factors model and radiomics signature, within most reasonable threshold probabilities. Figure 5 shows MRI and CTA images in representative patients with IPH and without IPH and nomogram results in these patients.

## Discussion

The detection of IPH is difficult by conventional CT imaging. In this study, we developed and validated a CT-based radiomics nomogram which incorporates a radiomics signature and clinical factor for the detection of intraplaque hemorrhage in carotid arteries. A CT-based radiomics nomogram demonstrated 84.2% accuracy, 88.9% sensitivity, and 80.0% specificity in distinguishing carotid plaques with or without intraplaque hemorrhage in an external test set. The CT-based radiomics nomogram showed better diagnostic performance than the clinical factor model in the external test sets (*p* = 0.039).

Several studies demonstrated that IPH is one of the most powerful predictors of cardiovascular event [14, 15]. If the IPH occurs, the treatment approach of the patient is significantly altered [16, 17]. Previous studies have tried to differentiate carotid plaques with IPH and those without IPH by using stenosis [18]. Several studies found that plaques with IPH in the carotid artery showed greater stenosis than those without IPH [15, 18], which are consistent with our studies. The reason may be that the stenosis of the lumen affected hemodynamic parameters, which in turn affected the status of intraplaque neovascularization. And, most investigators think that the rupture of immature neovessels may be the pathological basis of IPH [15], thus linking luminal stenosis with IPH. In this study, the degree of luminal stenosis was finally found as independent predictor, and the AUC of the clinical factor model was 0.631 for differentiating plaques with IPH and without IPH.

Currently, MRI is considered to be the best modality to detect the IPH in carotid arteries because the occurrence of IPH depends on the oxidate status of hemoglobin and it can be easily detected by common imaging sequences [19, 20]. However, MRI is not suitable for patients with metal stent and claustrophobia. CTA is a widely quick and convenient imaging modality for the pretreatment evaluation of patients with atherosclerosis due to its fast scanning speed, high spatial resolution, and accessibility [21]. Although several studies have shown that IPH shows a mean density of about 97.5 HU in CT, it is still difficult to differentiate IPH and lipid-rich necrotic core [22]. Therefore, the detection of IPH is difficult by conventional CT imaging and more dependent on the experience of radiologists.

Radiomics is an emerging technique that can automatically extract quantitative features from medical images [7, 8, 23]. Some researches about radiomics in oncology have been showed in predictions of the stages of tumors, differentiation between benign and malignant tumors, and predictions survival in patients with cancer [24, 25]. Radiomics research in the cardiovascular imaging has lagged behind the oncology field. The previous studies have investigated the prediction of thoracic aortic dissections with CT-based radiomics analysis [26]. Recent studies have shown the CT- and MRI-based radiomics analysis can accurately distinguish symptomatic from asymptomatic carotid and basilar plaques [9–11]. And several studies demonstrated that plaque texture analysis of ultrasound can identify the vulnerable carotid plaques and predict future ischemic events in asymptomatic patients [27, 28].

To our knowledge, our study is first to detect the IPH in carotid arteries by CT-based radiomics analysis. To date, several studies have considered IPH to be caused by the rupture of immature neoangiogenesis [29]. And IPH has been shown to promote plaque progression and instability, thereby increasing the risk of cerebrovascular events [30]. In addition, previous findings have demonstrated that the use of antithrombotic therapy may increase the progression of IPH in carotid plaques [16, 17]. Therefore, it is of great significance that our radiomics research makes up for the deficiency that conventional CT images cannot accurately recognize IPH in carotid arteries.

In this study, we constructed a CT-based radiomics nomogram, which showed satisfactory predictive efficacy with good calibration. In addition, more net benefit could be obtained from the decision curve analysis for most of the threshold probability model, implying that using our nomogram to identify carotid IPH would achieve better clinical outcomes. Secondly, three-dimensional ROI was performed in for our study. It is showed that three-dimensional radiomics analysis indicated heterogeneity better than two-dimensional ROI [31, 32]. Furthermore, unlike other single-center studies, the data in this study come from different institutions, which can yield more trustworthy results.

There are several limitations in our study. First, this is a retrospective study. But our finding warrants future prospective studies of predicting stroke risk after IPH by radiomics nomogram. Second, histological validation of IPH remains the goal standard, but our study has only a few with pathological validation that not all. However, MRI is currently the best in vivo image modality for IPH with high specificity and sensitivity. High-resolution MRI was available for each plaque in our study. Third, the quality of scans is dependent on the equipment manufacturer. Different equipment manufacturer in different hospitals can lead to inconsistencies in image quality. However, in order to eliminate the influence of dimension between features and make the intensity information consistent, the image was normalized before analysis in our study, which eliminated the interference caused by the inconsistent image quality caused by different CT equipment manufactures.

In conclusion, our study presented a CT-based nomogram that showed satisfactory performance in distinguishing carotid plaques with and without intraplaque hemorrhage. The radiomics nomogram may act as a non-invasive and potential tool to identify carotid intraplaque hemorrhage and make risk stratification.

## Supplementary Information

**Additional file 1**. Supplementary tables and figures.

## Data Availability

The data used and analyzed during the current study are available from the corresponding author on reasonable request.

## Abbreviations

ANOVA: Analysis of variance
AUC: Area under the curve
CI: Confidence intervals
DCA: Decision curve analysis
HU: Hounsfield units
ICC: Intraclass correlation coefficient
IPH: Intraplaque hemorrhage
LASSO: Least absolute shrinkage and selection operator
OR: Odds ratios
ROC: Receiver operator characteristic
ROI: Regions of interests
