# Supplementary material for: Radiomics assessment of carotid intraplaque hemorrhage: detecting the vulnerable patients
Source: Insights Imaging. 2022 Dec 20;13:200. doi: 10.1186/s13244-022-01324-2 (PMC9768061; doi:10.1186/s13244-022-01324-2)
Supplement: Supplementary file 1 — Additional file 1. Supplementary tables and figures. [file 13244_2022_1324_MOESM1_ESM.pdf]

ELECTRONIC SUPPLEMENTARY MATERIAL

Radiomics Assessment of Carotid Intraplaque Hemorrhage: Detecting the Vulnerable Patients

Supplementary Table S1 The MRI scanning parameters

Setting	Ingenia 3.0T	Prisma 3.0T	Verio 3.0T
Sequence	3D-T1-VISTA	3D-T1-SPACE	3D-T1-SPACE
Repetition time (ms)	425	700	700
Echo time (ms)	20	14	20
Field of view (mm)	140×140	160×160	140×140
Acquisition matrix	256×256	256×256	256×256
Slice thickness (mm)	1	0.6	1
Slice gap (mm)	-0.5	-1	0
Voxel size (mm^3)	0.7×0.7×0.7	0.6×0.6×0.6	0.9×0.9×0.9

3D = three dimensional; VISTA = volumetric isotropic turbo spin echo acquisition.

Supplementary Table S2 The more details of the radiomics features.

**Number**	**radiomics feature**
1	InterquartileRange
2	Skewness
3	Uniformity
4	Median
5	Energy
6	RobustMeanAbsoluteDeviation
7	MeanAbsoluteDeviation
8	TotalEnergy
9	Maximum
10	RootMeanSquared
11	90Percentile
12	Minimum
13	Entropy
14	Range
15	Variance
16	10Percentile

17	Kurtosis
18	Mean
19	VoxelVolume
20	Maximum3DDiameter
21	MeshVolume
22	MajorAxisLength
23	Sphericity
24	LeastAxisLength
25	Elongation
26	SurfaceVolumeRatio
27	Maximum2DDiameterSlice
28	Flatness
29	SurfaceArea
30	MinorAxisLength
31	Maximum2DDiameterColumn
32	Maximum2DDiameterRow
33	JointAverage
34	SumAverage
35	JointEntropy
36	ClusterShade
37	MaximumProbability
38	Idmn
39	JointEnergy
40	Contrast
41	DifferenceEntropy
42	InverseVariance
43	DifferenceVariance
44	Idn
45	Idm
46	Correlation
47	Autocorrelation
48	SumEntropy
49	MCC
50	SumSquares
51	ClusterProminence
52	Imc2
53	Imc1
54	DifferenceAverage
55	Id
56	ClusterTendency
57	GrayLevelVariance
58	HighGrayLevelEmphasis
59	DependenceEntropy

60	DependenceNonUniformity
61	GrayLevelNonUniformity
62	SmallDependenceEmphasis
63	SmallDependenceHighGrayLevelEmphasis
64	DependenceNonUniformityNormalized
65	LargeDependenceEmphasis
66	LargeDependenceLowGrayLevelEmphasis
67	DependenceVariance
68	LargeDependenceHighGrayLevelEmphasis
69	SmallDependenceLowGrayLevelEmphasis
70	LowGrayLevelEmphasis
71	ShortRunLowGrayLevelEmphasis
72	GrayLevelVariance
73	LowGrayLevelRunEmphasis
74	GrayLevelNonUniformityNormalized
75	RunVariance
76	GrayLevelNonUniformity
77	LongRunEmphasis
78	ShortRunHighGrayLevelEmphasis
79	RunLengthNonUniformity
80	ShortRunEmphasis
81	LongRunHighGrayLevelEmphasis
82	RunPercentage
83	LongRunLowGrayLevelEmphasis
84	RunEntropy
85	HighGrayLevelRunEmphasis
86	RunLengthNonUniformityNormalized
87	GrayLevelVariance
88	ZoneVariance
89	GrayLevelNonUniformityNormalized
90	SizeZoneNonUniformityNormalized
91	SizeZoneNonUniformity
92	GrayLevelNonUniformity
93	LargeAreaEmphasis
94	SmallAreaHighGrayLevelEmphasis
95	ZonePercentage
96	LargeAreaLowGrayLevelEmphasis
97	LargeAreaHighGrayLevelEmphasis
98	HighGrayLevelZoneEmphasis
99	SmallAreaEmphasis
100	LowGrayLevelZoneEmphasis
101	ZoneEntropy
102	SmallAreaLowGrayLevelEmphasis

103	Coarseness
104	Complexity
105	Strength
106	Contrast
107	Busyness
108	InterquartileRange
109	Skewness
110	Uniformity
111	Median
112	Energy
113	RobustMeanAbsoluteDeviation
114	MeanAbsoluteDeviation
115	TotalEnergy
116	Maximum
117	RootMeanSquared
118	90Percentile
119	Minimum
120	Entropy
121	Range
122	Variance
123	10Percentile
124	Kurtosis
125	Mean
126	JointAverage
127	SumAverage
128	JointEntropy
129	ClusterShade
130	MaximumProbability
131	Idmn
132	JointEnergy
133	Contrast
134	DifferenceEntropy
135	InverseVariance
136	DifferenceVariance
137	Idn
138	Idm
139	Correlation
140	Autocorrelation
141	SumEntropy
142	MCC
143	SumSquares
144	ClusterProminence
145	Imc2

146	Imc1
147	DifferenceAverage
148	Id
149	ClusterTendency
150	GrayLevelVariance
151	HighGrayLevelEmphasis
152	DependenceEntropy
153	DependenceNonUniformity
154	GrayLevelNonUniformity
155	SmallDependenceEmphasis
156	SmallDependenceHighGrayLevelEmphasis
157	DependenceNonUniformityNormalized
158	LargeDependenceEmphasis
159	LargeDependenceLowGrayLevelEmphasis
160	DependenceVariance
161	LargeDependenceHighGrayLevelEmphasis
162	SmallDependenceLowGrayLevelEmphasis
163	LowGrayLevelEmphasis
164	ShortRunLowGrayLevelEmphasis
165	GrayLevelVariance
166	LowGrayLevelRunEmphasis
167	GrayLevelNonUniformityNormalized
168	RunVariance
169	GrayLevelNonUniformity
170	LongRunEmphasis
171	ShortRunHighGrayLevelEmphasis
172	RunLengthNonUniformity
173	ShortRunEmphasis
174	LongRunHighGrayLevelEmphasis
175	RunPercentage
176	LongRunLowGrayLevelEmphasis
177	RunEntropy
178	HighGrayLevelRunEmphasis
179	RunLengthNonUniformityNormalized
180	GrayLevelVariance
181	ZoneVariance
182	GrayLevelNonUniformityNormalized
183	SizeZoneNonUniformityNormalized
184	SizeZoneNonUniformity
185	GrayLevelNonUniformity
186	LargeAreaEmphasis
187	SmallAreaHighGrayLevelEmphasis
188	ZonePercentage

189	LargeAreaLowGrayLevelEmphasis
190	LargeAreaHighGrayLevelEmphasis
191	HighGrayLevelZoneEmphasis
192	SmallAreaEmphasis
193	LowGrayLevelZoneEmphasis
194	ZoneEntropy
195	SmallAreaLowGrayLevelEmphasis
196	Coarseness
197	Complexity
198	Strength
199	Contrast
200	Busyness
201	InterquartileRange
202	Skewness
203	Uniformity
204	Median
205	Energy
206	RobustMeanAbsoluteDeviation
207	MeanAbsoluteDeviation
208	TotalEnergy
209	Maximum
210	RootMeanSquared
211	90Percentile
212	Minimum
213	Entropy
214	Range
215	Variance
216	10Percentile
217	Kurtosis
218	Mean
219	JointAverage
220	SumAverage
221	JointEntropy
222	ClusterShade
223	MaximumProbability
224	Idmn
225	JointEnergy
226	Contrast
227	DifferenceEntropy
228	InverseVariance
229	DifferenceVariance
230	Idn
231	Idm

232	Correlation
233	Autocorrelation
234	SumEntropy

Supplementary Figure 1.

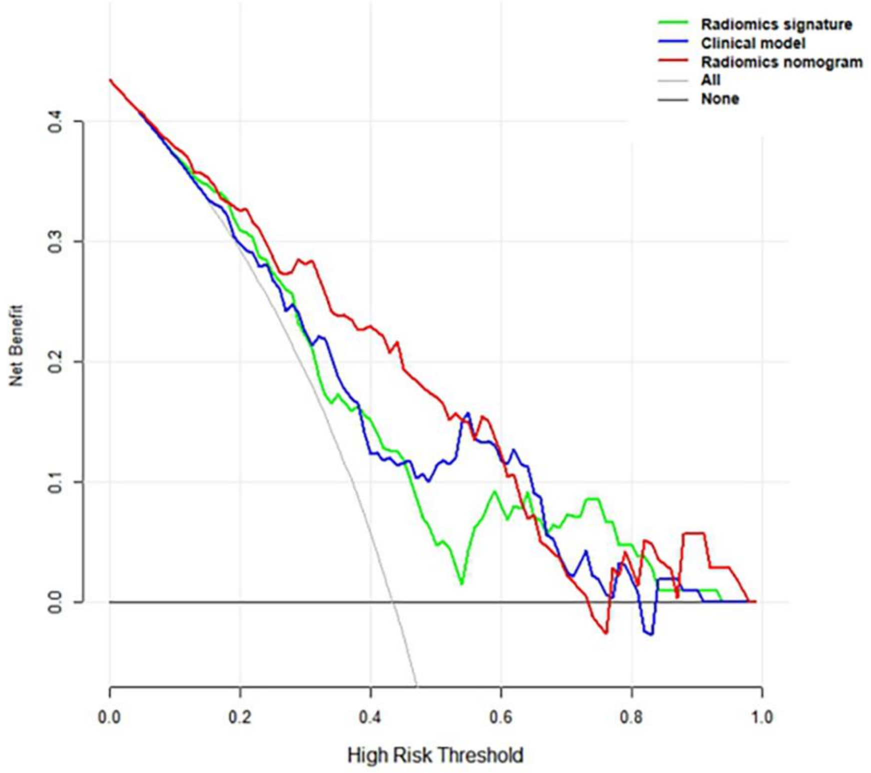

Decision curve analysis for three models. The y-axis indicates the net benefit; x-axis indicates threshold probability. The blue line, green line, and red line represent net benefit of the clinical factor, the radiomics signature, and the radiomics nomogram, respectively. The gray line indicates the hypothesis that all patients had carotid plaques with IPH. The black line indicates the hypothesis that no patients had carotid plaques with IPH. The decision curves indicate that the application of radiomics nomogram to predict carotid plaques with IPH adds more benefit than two models,

and treating all or none of the patients, across the full range of threshold probabilities.

IPH = intraplaque hemorrhage.
